# RNAPattMatch: a web server for RNA sequence/structure motif detection based on pattern matching with flexible gaps

**DOI:** 10.1093/nar/gkv435

**Published:** 2015-05-04

**Authors:** Matan Drory Retwitzer, Maya Polishchuk, Elena Churkin, Ilona Kifer, Zohar Yakhini, Danny Barash

**Affiliations:** 1 Department of Computer Science, Ben-Gurion University, Beer-Sheva 84105, Israel; 2 Department of Computational Biology, Vavilov Institute of General Genetics, Russian Academy of Science, Moscow 11933, Russia; 3 Agilent Laboratories, Agilent Technologies, Tel Aviv 49527, Israel; 4 Microsoft R&D Center, Herzliya 46725 , Israel; 5 Laboratory of Computational Biology, Computer Science Department, Israel Institute of Technology, Haifa 32000, Israel

## Abstract

Searching for RNA sequence-structure patterns is becoming an essential tool for RNA practitioners. Novel discoveries of regulatory non-coding RNAs in targeted organisms and the motivation to find them across a wide range of organisms have prompted the use of computational RNA pattern matching as an enhancement to sequence similarity. State-of-the-art programs differ by the flexibility of patterns allowed as queries and by their simplicity of use. In particular—no existing method is available as a user-friendly web server. A general program that searches for RNA sequence-structure patterns is RNA Structator. However, it is not available as a web server and does not provide the option to allow flexible gap pattern representation with an upper bound of the gap length being specified at any position in the sequence. Here, we introduce RNAPattMatch, a web-based application that is user friendly and makes sequence/structure RNA queries accessible to practitioners of various background and proficiency. It also extends RNA Structator and allows a more flexible variable gaps representation, in addition to analysis of results using energy minimization methods. RNAPattMatch service is available at http://www.cs.bgu.ac.il/rnapattmatch. A standalone version of the search tool is also available to download at the site.

## INTRODUCTION

The search for homology of biological molecules is considered an important endeavor in the field of bioinformatics. In particular, searching genomes for peculiar RNAs such as ribozymes and riboswitches (1), as well as other examples of RNA molecules that can possess catalytic roles (2) or non-coding RNAs that can participate as regulators of disease (3), is undoubtedly an indispensable computational task. Homology in sequence is often not enough for a more comprehensive detection and RNA homology search methods that integrate what is known about the secondary structure of functional RNAs should have a higher specificity and sensitivity. For example, a simple pattern matching approach that utilizes RNA sequence-structure patterns was used to find the first eukaryotic riboswitches in fungi and plants (4). Finding additional eukaryotic riboswitches in new organisms besides fungi and plants (e.g. in animals) is still one of the main open questions in riboswitch research that extends beyond the many already discovered prokaryotic riboswitches (5,6). Another example of the use of sequence-structure patterns is in the search for tRNAs (7,8). The search methods can vary in sophistication, some may use experimental evidence for the secondary structure and others may also combine secondary structure predictions by energy minimization (9,10), as was attempted for riboswitch identification in (11,12). We note that there are also more elaborate methods to search for riboswitches and other non-coding RNAs in genomes (13–15) but pattern matching remains a simple and useful approach.

The Denison Riboswitch Detector (DRD) (12) follows other riboswitch detectors that utilize pattern matching (16–18). All of these riboswitch detectors are available as web servers. However, except for the DRD, all other riboswitch detectors contain known riboswitches and do not allow a flexible definition file of a new riboswitch to be inserted by the user. Even for DRD, inserting a new sequence-structure pattern for which an experimentally derived structure is available is a complicated task for a less experienced practitioner. The definition file expects some parameters such as the minimum number of identities in a global alignment of Vienna strings (strings that compromise of dots and brackets for RNA secondary structure representation (10)) that are not straight-forward to derive for simple sequence-structure patterns as available in (4,19–20). Moreover, DRD considers mismatches, a strategy that was never attempted to be considered in the simple yet biologically significant pattern search programs used in (4,19–20) because mismatches will ultimately result in more solutions, some of them spurious, instead of changing the sequence-structure pattern to become more flexible in order to obtain more solutions in a biologically meaningful way. In our opinion, it is more important to consider variable gaps than mismatches and also to opt for a user-friendly web server in which the practitioner can insert simple sequence-structure patterns without some added complications.

Several programs that are not available as web servers have been developed over the years, implementing the RNA pattern matching approach. They range in their content sophistication from simple and specific ones, like the SequenceSniffer used in (4,19) and the RNA-PATTERN used in (20) in the context of riboswitches, to more complicated and general ones like RNAmot (21), RNAbob from the Eddy/Rivas lab, PatSearch (22), RNAmotif (23) and Structator (24). These general-purpose programs are also more sophisticated for the practitioner. When developing the RNAPattMatch web server, we aimed at reaching a user-friendly server that contains the most important ingredients of a general-purpose program and yet being simple for users of different backgrounds, practical and efficient. From the methodology standpoint, closest to our approach is the RNA Structator. Analogous to the Structator (24), we utilize the highly efficient index data structure called affix arrays that is suitable for sequence-structure patterns. The affix array data structure is equivalent to the affix tree with respect to its algorithmic functionality for pattern matching but with smaller memory requirements and improved performance (25). However, by allowing more flexible variable gaps (providing an upper bound of the gap length permitted at any position in the sequence) as part of the pattern definition, as is also becoming available in *de novo* motif search tools beyond RNAs (26), and by analyzing the results using energy-minimization methods, we extend the capabilities of the Structator, in addition to providing a user-friendly web server.

To address the need for a sequence/structure pattern matching web server, which is flexible in terms of patterns it allows to search for and which is user friendly and simple, we have now developed RNAPattMatch. As summarized in a broader context in the next paragraph, the ability of RNAPattMatch to search multiple sequences in a single run is a considerable support to its biological significance, since it can provide a convincing substitute to in-house programs such as SequenceSniffer (4) and RNA-PATTERN (20), which are not trivial to develop in typical biology labs. Not all experimental labs have the resources to write such in-house programs and our web server is geared toward providing a convenient and efficient answer to the needs of such biology labs who would like to perform a pattern search. The user can upload FASTA files of up to 100 MB that consist of different sequences. For example, RNAPattMatch can scan around a hundred bacterial genomic sequences as performed in (20) by uploading one or more target files in FASTA format (most bacterial genomes are under 10 MB, e.g. the complete genome of *Thermoanaerobacteria tengcongensis* MB4 that is illustrated in the figures is 2.6 MB) and then searching the multiple sequences in a single or very few runs. In another example, the user can receive a FASTA file containing BLAST results as performed in (4) and search for the same pattern using RNAPattMatch in a user-friendly manner.

To summarize and put more generally, one distinctive feature of RNAPattMatch is the fact it accepts FASTA files as target sequences for the search. This allows for flexible usage in several contexts such as: search pattern in multiple genomes (as done in (20)), in transcriptomics data actually measured in metagenome samples, in lists of differentially expressed transcripts or such that were determined, by measurement, to have some other molecular property such as binding an RNA-binding protein (RBP) (27). These directions also represent future research directions that will efficiently utilize the computational power of RNAPattMatch.

## PATTMATCH WEB SERVER

The RNAPattMatch web server (http://www.cs.bgu.ac.il/rnapattmatch) runs on a Unix IBM x3550 M4 server with Quad Intel(R) Xeon(R) CPU E5-2620 2.00GHz processors containing six logical cores and 15 MB L3 cache each. Memory size is 64 GB to allow for the extensive memory needed for the affix arrays data structure. Every search task runs on up to four cores depending on load. The server runs up to 10 simultaneous search tasks while the rest wait in a queue.

### Input

**Figure 1.**
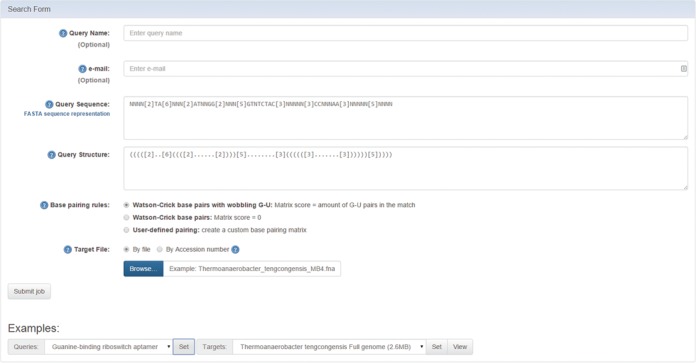
The input screen of the RNAPattMatch web server with the example sequence-structure pattern query taken from (19), a target file of 2.6MB containing the complete genome of *Thermoanaerobacteria tengcongensis* MB4 and example parameters inserted. The target file (up to 100 MB) may contain several genome sequences in FASTA format.

The input screen of the RNAPattMatch web server is shown in Figure 1. Initially, the user provides a query combining a pattern of RNA sequence and a pattern of RNA structure. The sequence pattern supports FASTA nucleic acid codes, as well as a variable gap represented by ‘[*x*]’ that accounts for up to *x* nucleotides. The structure pattern supports the dot-bracket notation for secondary structure representation (matching parentheses denote paired bases and dots denote free bases), as well as a variable gap represented by ‘[*x*]’ (same as in corresponding sequence pattern) that accounts for up to *x* nucleotides. In addition, the user provides a cost matrix for the various base-pairing interactions. The matrix will show by pressing the ‘user defined pairing’ option, otherwise it will allow for default matrix that supports Watson–Crick pairing with G-U pairing. Finally, the user provides a target sequence. The target sequence can be either a file (up to 100 MB, in FASTA format) that consists of nucleic acid codes or a GenBank (28) accession number containing a nucleic acid sequence. Wild card codes in the target such as ‘N’ will mismatch any query character. Optional fields are the email address of the user and query name that can be used to locate the job later on. If the user specifies an email address, the results will arrive by email, otherwise the results will be available in an interactive job mode. Aside of the ‘Find Pattern’ page, a ‘Search Result’ page is available in the top menu, should the user wish to find a result that was computed already using its corresponding query name or query identification. A general ‘Help’ page is also available, as well as the tooltips that provide some brief help explanations for each field.

### Output

The results are guaranteed to be kept for at least one week after they are generated in the web link that is provided to the user. In addition to keeping the web link for later use, the user has an option to download the results in excel format for further analysis.

**Figure 2.**
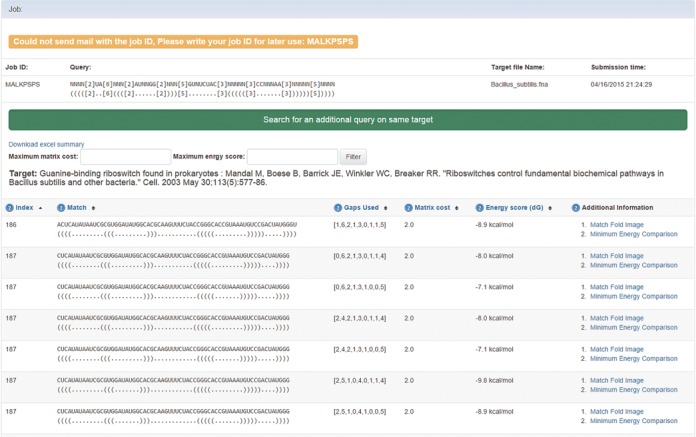
The results screen of the RNAPattMatch web server, matches are found in a table with options to sort and filter by selected parameters. Each row provides a match result by specifying its start index in the target, the corresponding sequence from the target and its aligned structure including the gaps used for the specific match, the amount of gaps used for the match, the matrix cost, the energy of the given match and an option to view the predicted secondary structure drawing while comparing it with the secondary structure drawing of the match itself. There is an option to create an additional query on the same file. If the caching of the data structure is completed it will be used.

**Figure 3.**
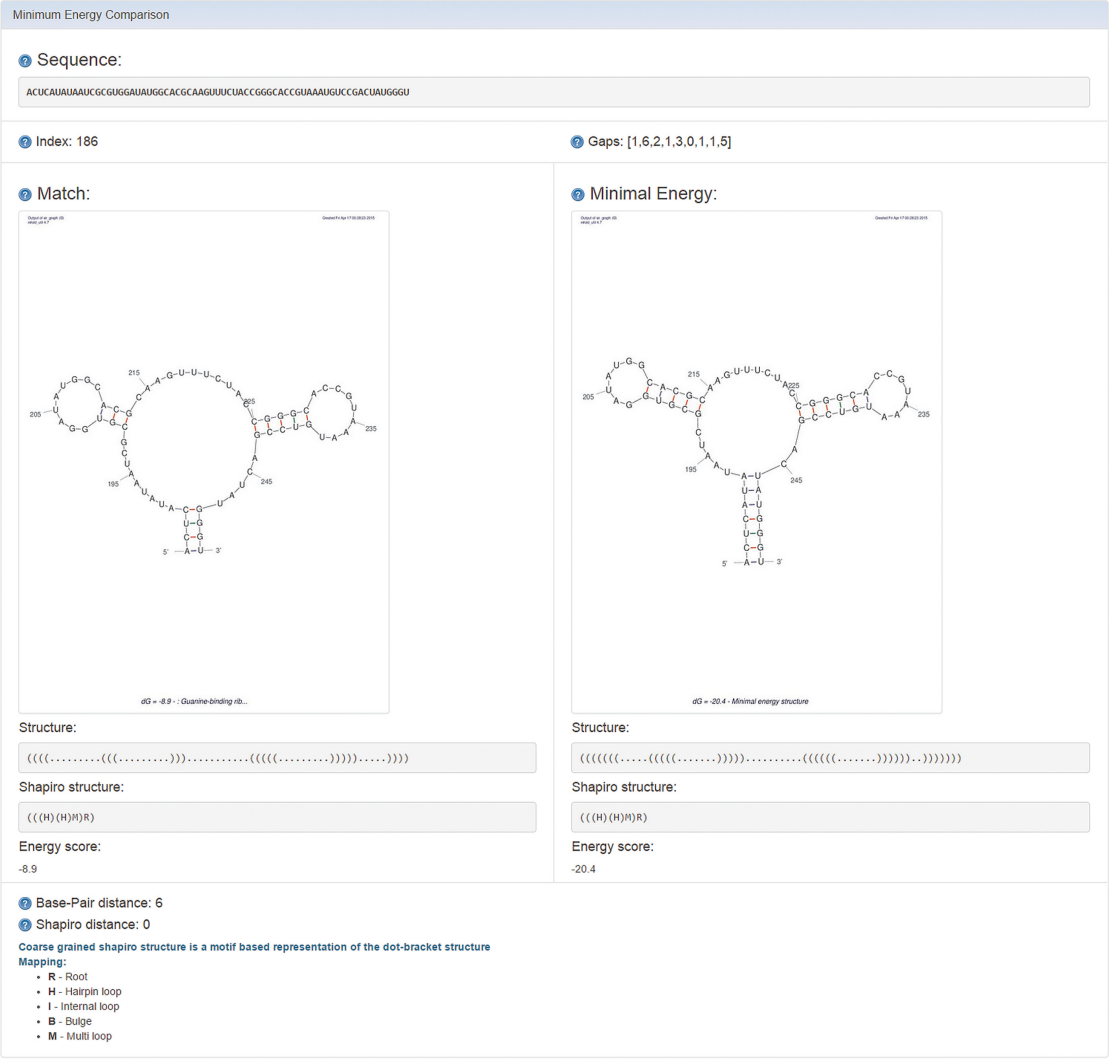
The predicted secondary structure of the first found match using Mfold (9) for the secondary structure drawing, while comparing it with the secondary structure drawing of the match itself. Secondary structures are represented by Vienna's dot-bracket representation (10) and the coarse-grained Shapiro representation (30). As expected from the pattern query, the structure drawings correspond to the purine riboswitch aptamer described in (19).

After the example parameters in the input screen of Figure 1 are inserted and the form is submitted, the main results screen appearing in Figure 2 is obtained. The query sequence-structure pattern appears at the top of the page with a link to generate an additional query for the same target file. Below it is a table with a list of results. The table contains all matching sequences with an option to sort and filter by selected parameters. Each row provides a match result by specifying its start index in the target, the corresponding sequence from the target and its aligned structure including the gaps used for the specific match, the amount of gaps used for the match, the matrix cost, the energy of the given match in kcal/mol (calculated using RNAeval from the Vienna RNA package (10), according to the Turner energy model, 2004 (29)) and an option to view the predicted secondary structure drawing using sir_graph of Mfold (9). The user can click on ‘Match Fold Image’ in each row, and a popup window will show the secondary structure of the match. Another option is the ‘Minimum Energy Comparison’ that shows both the secondary structure of the match and the predicted minimum energy structure of the matched sequence (calculated by RNAfold from the Vienna RNA package (10)) and the distance in secondary structure. Figure 3 depicts the predicted secondary structure of the first found match. Secondary structures are represented by Vienna's dot-bracket representation (10) and the coarse-grained Shapiro representation (30). As expected from the pattern query in the example, the structure drawing corresponds to the purine riboswitch aptamer described in (19).

## THE PATTMATCH METHOD

Historically, many data structures were developed in order to solve pattern matching in text. Suffix trees are indexed data structures that allow for fast pattern matching. Each path from the root to a leaf in the tree outlines a suffix of the target sequence while each branch is a diversion between suffixes with different letters at the position of the height of that vertex. Searches based on this method compare each letter from the query while progressing down the tree, such that once all the query was compared, the entire sub tree of that given vertex are occurrences of the query. Since suffix trees are a memory consuming data structure, most modern tools use a more compact version called suffix array or enhanced suffix array. Enhanced suffix arrays introduce a few additions that allow for faster pattern matching. The longest common prefix is computed for each suffix compared to the suffix before it, and the child table gives us the ability to quickly find an interval of suffixes with longer common prefixes within the interval, similar to walking down a suffix tree. This data structure can perform fast pattern matching in an unidirectional search which progresses along a given query from start to end.

The problem of RNA sequence-structure pattern matching introduces additional information not used in a search based on suffix arrays. The unidirectional search runs over the entire query string from start to end, thus ignoring the additional information regarding base-pairing until it reaches the closing base. For this specific reason the affix tree, or its more compact version the affix array, were introduced. The affix array uses a suffix array in conjunction with a reverse prefix array, which can be described as the suffix array of the reverse target string and an additional set of links between them. Those affix links connect an interval from the suffix array to an interval in the reverse prefix array, such that the longest common prefix representing the suffix interval is equal to the end of the longest common suffix of the interval in the reverse prefix array. Similar links are connected in the reverse direction. This new data structure allows us to perform a bidirectional search, such that the order of comparison tests the closing base right after comparing the opening base which reduces the amount of intervals we need to test, especially when comparing wild cards like the ‘N’ letter in the FASTA format. The bidirectional search supports very fast and efficient matching of hairpin-like motifs. Generating the data structure is a large task, which is why we only calculate affix links when they are needed by the search algorithm. In order to search for more complex structures we break the query structure into multiple hairpin-like sections. Each section, starting from the most specific which includes the least wild card FASTA letters, is matched using the bidirectional search. Once a hairpin search is done, we then merge the matches with the hairpins we found before it. The merging process includes sorting the new hairpin matches by the index facing the hairpins we found before it and checking which new matches are at the correct distance. Once a full set of hairpins has been assembled we compare the rest of the non-hairpin sections to the query. We refer readers who are interested in the bidirectional search scheme to reference (25).

Our web server sends search requests to an underlying tool implemented in C++ that performs efficient pattern matching using a dynamic programming based search that solves generated tasks regarding an interval of sequences and a sub-query, such that the interval fits the query up to the sub-query. The tasks generated take into account the positions of variable gaps inserted by the user. The tool's source code can be downloaded at the website under the Additional Information tab. Our web server dispatches up to 10 simultaneous instances of the search tool, one for each job. Additionally for each new target sequence, the server starts another instance that generates the full data structure and saves it in a file. This caching mechanism is then later used in additional queries of that same target. Run times of the example queries on several target files can be viewed in Table 1.

**Table 1:** Running times for the example queries on selected target files

Query	*Thermoanaerobacter tengcongensis* MB4 (2.7 mb)	*Saccharomyces cerevisiae* sacCer3 (12mb)	Human chromosome 16 hg38, GRCh38 (89mb)
Guanine-binding riboswitch aptamer^a^	2(s)^b^	6(s)	127(s)
Hairpin with G-C stem^c^	7(s)	29(s)	146(s)

^a^Matches for guanine riboswitch were not found in the non-bacterial organisms reported in the table.

^b^Running times were taken from the RNAPattMatch web server on non-cached targets and do not include file upload time.

^c^Difficulty of the query pattern is dictated by the amount of hairpins and the size of their search space as observed in (25).

Query sequences are available as examples in the web server.

## CONCLUSIONS

When searching for homology of RNAs in genomes, sequence considerations alone are limited and finding sequence-structure patterns offers an improvement in case such a pattern is known in advance. Indeed, such patterns are available in practice, e.g. (4,20), and there is a clear biological motivation for their search. Several programs have been developed over the years to find sequence-structure patterns, but none are available as a user-friendly web server that can accommodate practitioners of various backgrounds. We present a new web server called RNAPattMatch that fulfills this need. It is based on the methodology implemented in the program (not available as a web server) called RNA Structator (24) and offers a significant extension to Structator, by addressing variable gaps and providing a comprehensive analysis of results with RNA folding prediction by energy minimization including secondary structure drawings.

The RNAPattMatch web server was developed with the goal of making the efficient method of using affix arrays and a dynamic programming search algorithm available for the entire biological community. The web server is user-friendly and accessible to practitioners, both in terms of ease of use and simplification of the output. We believe that it will serve experimental groups for improving their capability to perform RNA sequence-structure pattern search.
